# The Big Five Personality Factors Among Cybercrime Victims in Jordan in Light of Some Variables

**DOI:** 10.1192/j.eurpsy.2025.1795

**Published:** 2025-08-26

**Authors:** S. F. Abu Dayeh, A. Khawaldeh, B. Shawqfeh

**Affiliations:** 1 psychiatry; 2 psychology, moh; 3 psychology, jordan university, amman, Jordan

## Abstract

**Introduction:**

This study aimed to identify the big five personality factors among cybercrime victims in Jordan and to investigate the differences in the level of the big five personality factors due to the type of crime, gender, age, and educational level. The descriptive analytical approach was used. The study sample consisted of (515) cybercrime victims in Jordan. To achieve the aims of the study, the five major factor scale was used, and its reliability and validity were checked.

**Objectives:**

Study Objectives:

1. ersonality traits of cybercrime victims in Jordan.
2. The differences in the Big Five personality traits of cybercrime victims based on the type of cybercrime.
3. whether Big Five personality traits among cybercrime victims varied significantly with regard to gender, age, and educational status of respondents.

**Methods:**

**Study Tool: The Big Five Personality Traits Scale**

This study used The Big Five Personality Traits Scale (Costa & McCrae, 1992) . In its original configuration, the scale consisted of 60 items or questions.

Item Characteristics of the Assessments Scale: 1.Internal Construct Validity. Internal consistency was checked using Cronbach’s alpha. The Cronbach’s alpha reliability coefficient was 0.701to 0.818, which was above 0.70; therefore, it is acceptable. Scoring of the scale: A 5-point Likert scale was employed .The second question was approached with One-way ANOVA. The third question was addressed via MANOVA.

**Results:**

The results show that the level of extraversion and openness factors came at a high level, while the conscientiousness, agreeableness, and neuroticism factors came at a moderate level. Also, the results found that there were no differences due to the type of crime in the neuroticism factor and there were differences in the factors (extraversion in favor of device hacking crime, openness in favor of unwanted sexual crime, conscientiousness, and agreeableness in favor of identity theft crime). Also, the results show that there are differences in the factors (neuroticism, extraversion, and openness) due to gender in favor of females, and due to age in the factors (neuroticism, acceptability, and conscientiousness) in favor of less than 30 years, and the level of education in the openness factor in favor of a secondary class or less, while in the acceptability factor in favor of a bachelor, while in the scientific factor in favor of postgraduate students.

**Conclusions:**

Actions should be taken against cybercrimes, which include:

- More education about cybercrimes and how to use internet in a safe way.
- Supervision of people who are still not efficient in using technology and close supervision of the way that teenagers are using high technology devices.
- Trainings should be done to start protecting people from such crimes.

**Disclosure of Interest:**

None Declared

